# Clinical Prevalence, Antibiogram Profiling and Gompertz Growth Kinetics of Resistant *Staphylococcus epidermidis* Treated with Nanoparticles of Rosin Extracted from *Pinus roxburghii*

**DOI:** 10.3390/antibiotics11091270

**Published:** 2022-09-19

**Authors:** Zahid Majeed, Muhammad Qudir Javid, Shamyla Nawazish, Basharat Ahmad, Abu ul Hassan Faiz, Ayesha Baig, Sofia Baig, Mater H. Mahnashi, Naif A. Jalal, Abdulaziz Asiri, Amer Al Ali

**Affiliations:** 1 Department of Biotechnology, Faculty of Science, The University of Azad Jammu and Kashmir, Chehla Campus, Muzaffarabad 13100, Pakistan; 2 Department of Environmental Sciences, COMSATS University Islamabad, Abbottabad Campus, Abbottabad 22020, Pakistan; 3 Department of Zoology, Faculty of Science, The University of Azad Jammu and Kashmir, King Abdullah Campus, Muzaffarabad 13100, Pakistan; 4 Department of Zoology, Faculty of Science, Woman University of Azad Jammu and Kashmir, Bagh 12500, Pakistan; 5 Department of Biotechnology, COMSATS University Islamabad, Abbottabad Campus, Abbottabad 22020, Pakistan; 6 Institute of Environmental Sciences and Engineering (IESE), National University of Sciences and Technology (NUST), Islamabad 44000, Pakistan; 7 Department of Pharmaceutical Chemistry, College of Pharmacy, Najran University, Najran 66262, Saudi Arabia; 8 Department of Microbiology, Faculty of Medicine, Umm Al-Qura University, Makkah 21955, Saudi Arabia; 9 Faculty of Applied Medical Sciences, University of Bisha, 255, Al Nakhil, Bisha 67714, Saudi Arabia; 10 Department of Clinical Laboratory Sciences, Faculty of Applied Medical Sciences, University of Bisha, 255, Al Nakhil, Bisha 67714, Saudi Arabia

**Keywords:** drug resistance, *S. epidermidis*, antibiogram, prevalence, rosin maleic anhydride nanoparticles

## Abstract

The rise of methicillin-resistant *Staphylococcus epidermidis* (MRSE) makes it difficult to treat infections that increase morbidity and mortality rates in various parts of the world. The study’s objectives include identifying the clinical prevalence, antibiogram profile, and Gompertz growth kinetics of MRSE treated with synthetically created nanoparticles of rosin obtained from *Pinus roxburghii*. A total of 64 of 200 clinical isolates of *S. epidermidis* (32% of the total) displayed sensitivity (40.62%) and resistance (59.37%) to seven different antibiotic classes. The most sensitive patterns of antibiotic resistance were seen in 20 (78.95%) and 24 (94.74%) isolates of MRSE against piperacillin/tazobactam and cephradine, respectively. Fosfomycine was found to be the most effective antibiotic against MRSE in 34 (89.47%) isolates, followed by amoxicillin. Successfully produced, described, and used against MRSE were rosin maleic anhydride nanoparticles with a size range of 250 nm to 350 nm. Five different concentrations of 25, 50, 75, 100, and 150 mg mL^−1^ rosin maleic anhydride nanoparticles were investigated to treat MRSE resistance. According to Gompertz growth kinetics, the maximal growth response was 32.54% higher and the lag phase was also 10.26% longer compared to the control when the amount of rosin maleic anhydride nanoparticles was increased in the MRSE. Following the application of rosin maleic anhydride nanoparticles, the growth period is extended from 6 to 8 h. A potential mechanism for cell disintegration and distortion is put forth. This investigation came to the conclusion that rosin maleic anhydride nanoparticles better interfere with the surface of MRSE and demonstrated a preferred bacteriostatic action.

## 1. Introduction

The coagulase-negative, Gram-positive commensal *Staphylococcus epidermidis* (*S. epidermidis*) is a kind of *Staphylococcus*. One of the microorganisms that affect people the most is this one. The assessment of its antimicrobial susceptibility is given the least attention despite the fact that it carries the genes implicated in pathogenicity and is frequently isolated as a common contaminant in clinical samples [1]. It is a frequent source of infection that spreads via medical devices, skin, mucous membranes, and immunocompromised individuals [2,3,4]. The worldwide appearance and rapid spread of S. epidermidis’ multi-drug resistant lineages have been reported in recent communications [5]. As a result, the issue of *S. epidermidis*’ emergence and infection as a nosocomial infectious agent has raised awareness of the need to stop its global spread.

One of the biggest global public health issues is antibiotic resistance, according to the World Health Organization. Many microbiological species exposed to these antibiotics develop resistance as a result of the emergence of genetic mutations in genes implicated in antibiotic resistance pathways [6]. The evolution of resistance is mostly driven by the quick rate of reinfection by different strains that are resistant to the advised antibiotics, according to analyses of 140,349 infections of the urinary tract and 7365 infections originating from wounds [7].

Biofilms created by *S. epidermidis* aid in the organism’s adhesion to surfaces. These interactions with the surface are facilitated by a group of genes. The *S. epidermidis* bacteria’s *icaD* gene, which codes for N-acetyl glucosamine transferase, aids in the biofilm’s exopolysaccharide matrix synthesis [8]. The amount of expression of the elastin genes *FnbA* and EbpS has been observed, communicating the involvement of these genes in facilitating *S. epidermidis* pathogenicity [9]. Both in vivo and in vitro, fibronectin-binding protein (FnbA) contributes to *S. epidermidis’* enhanced capacity to infect endothelium cells [10]. When *S. epidermidis* colonizes tissue, its elastin-binding protein (EbpS) binds to the N-terminal region of elastin [11]. Antibiotics such as imipenem and vancomycin are frequently recommended to treat infections brought on by the drug-resistant *S. epidermidis*. Unfortunately, using the potentially dangerous glycol-peptide vancomycin to treat *S. epidermidis* resistance to methicillin has become commonplace. However, published research has shown that the emergence of intermediate vancomycin resistance in two strains, *S. epidermidis* and *S. haemolyticus*, is concerning. In order to find effective treatments for *Staphylococcus* infections, more research is needed due to the limited number of available drugs [12,13,14].

The stem of *Pinus* trees exudes rosin. Dehydroabietic, abietic, levopimaric, neoabietic, palustric, and isopimaric acids make up the majority of the hydrophobic diterpene carboxylic acids that make up rosin [15]. The confirmation of antibacterial effectiveness against numerous types of microbes was supplied by in vitro tests [16,17,18,19]. Skin sores and lesions have been seen to heal after rosin therapy [20,21]. The full description of rosin’s antibacterial mode of action is constrained by its non-homogeneous composition. However, methods such as transmission and scanning electron microscopy led researchers to conclude that rosin alters the thickness of the cell walls and impacts cell-to-cell adhesion, fatty acid content, and membrane potential in *S. aureus* [22,23], which caused energy-linked metabolic reactions related to the growth and physiology of the bacterial cell to be structurally and functionally interrupted [24]. There are many scientific fields where the study of nanoparticles has found use. Nanotechnology, in particular, changed the field of natural chemical antibacterial usage. Through the use of nanotechnology, naturally occurring materials with a lot of chemical functional groups have been used to create medications or drug delivery systems. Both internal and extrinsic parameters, such as pH, enzymatic reaction resistance, water activity, relative humidity, temperature, and storage environment, are influenced by nanoscale designs [25].

When utilized in vitro for bacterial research, rosin demonstrates active antibacterial activity against a wide range of pathogens [18]. The rosin-based salve demonstrated the healing of skin infections linked to ulcers and wounds in a clinical experiment. The interaction of *S. epidermidis* with rosin disrupted cell aggregation, wall thickness, membrane potential, and fatty acid structure, according to scanning electron microscopy, electron physiology, and transmission, even though the precise mechanism underlying rosin acid’s antimicrobial action is not yet fully understood. Bacteria lose their ability to maintain the integrity of their cell membrane as a result, which ultimately disrupts the metabolic process and cell viability [26].

Since the *S. epidermidis* bacterium is a multidrug-resistant Gram-positive organism, research on natural plant products for treating *S. epidermidis* infections may help to address the global issue of antibiotic resistance. The creation of rosin-based rosin maleic anhydride nanoparticles and the in vitro management of *S. epidermidis* infections resistant to conventional treatments are the two main areas of the study. This study was done to determine the prevalence of *S. epidermidis* isolates from various clinical samples taken from patients who visited the Combined Military Hospital (CMH), Muzaffarabad, Azad Kashmir, Pakistan indoors and outdoors. Aiming to test *S. epidermidis’* sensitivity, various antibiotics are used. To create rosin maleic anhydride nanoparticles, rosin is treated with maleic anhydride in order to reevaluate the susceptibility of the resistant *S. epidermidis* to these nanoparticle formulations. Using the empirical model equation of Gompertz kinetics and the tolerance of rosin maleic anhydride nanoparticles in clinical isolates, the rate of growth over time is evaluated. The goal of this research project is to investigate the clinical prevalence, antibiogram profiling, and Gompertz growth kinetics of resistant *S. epidermidis* that has been exposed to rosin maleic anhydride nanoparticles that were made from rosin derived from *Pinus roxburghii.*

## 2. Results

### 2.1. Prevalence and Antibiotics Sensitivity in S. epidermidis Isolates

**Figure 1:**
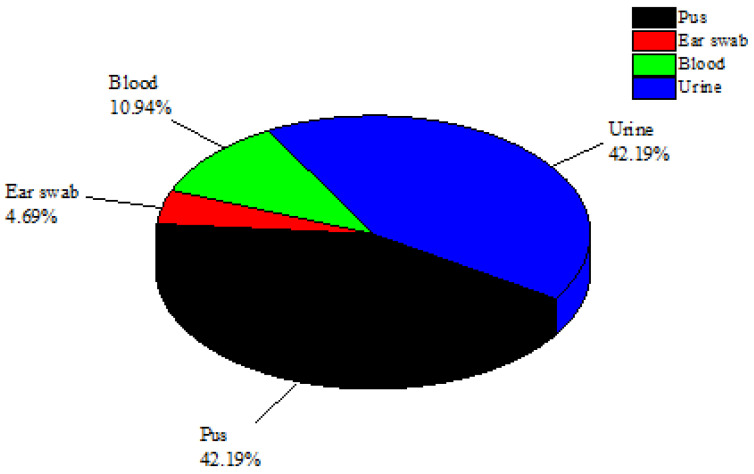
*S. epidermidis* prevalence (number and percentage) in various clinical infection samples.

From the CMH in Muzaffarabad, 200 clinical infectious samples were studied in total. Only 64 bacterial isolates from these 200 samples were identified as *S. epidermidis* bacteria. Only 27 of these 64 *S. epidermidis* isolates were found in urine samples (42.19%), while 27 isolates were found in pus samples (42.19%). Additionally, three *S. epidermidis* isolates were filtered out of ear swab samples and seven isolates of *S. epidermidis* were found in blood samples (10.94%) (Figure 1). The urine and pus samples were the most contagious clinical samples and were found to contain the greatest concentrations of *S. epidermidis*. The samples of blood and ear swabs, however, had the lowest *S. epidermidis* recovery rates. Through biochemical tests such as catalase and coagulase as well as genetic testing using 16sRNA sequencing, all isolates of *S. epidermidis* were verified.

In total, 64 *S. epidermidis* isolates were screened for MRSE and MSSE based on the types of samples and places where the specimens were collected (Table 1). Of them (N = 64), 38 recovered *S. epidermidis* isolates were MRSE and 26 were MSSE. The highest concentrations of MRSE and MSSE were found in urine and pus samples, respectively.

The sensitivity and resistance to several antibiotics were evaluated for *S. epidermidis* isolates (Table 2). The antibiotics piperacillin/tazobactam in 24 (94.74%) and cephradine in 20 (78.95%) isolates had the most sensitive patterns in MSSE based on the disc-diffusion approach. Fosfomycin, cefoxitin, and amoxicillin all showed greater levels of antibiotic resistance in MRSE samples, in that order: 34 (89.47%) isolates, 30 (78.95%) isolates, and 28 (73.68%) isolates.

### 2.2. Rosin Maleic Anhydride Nanoparticles

**Figure 2:**
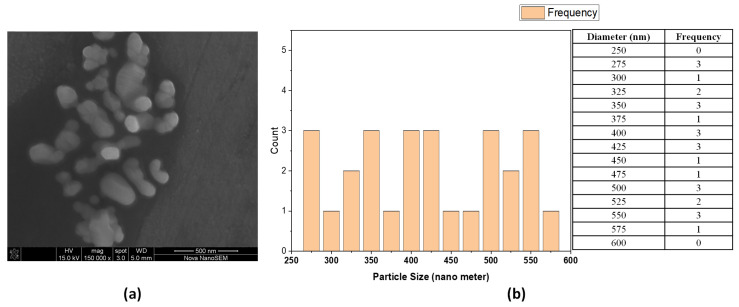
FESEM of rosin maleic anhydride nanoparticles: (**a**) Morphology, (**b**) distribution.

Rosin maleic anhydride nanoparticles’ microstructure was elucidated by high-resolution FESEM (Figure 2). Rosin maleic anhydride nanoparticles were successfully produced under experimental conditions. FESEM was successfully used to study the physical structure and configuration of the produced rosin maleic anhydride nanoparticles. The morphology of the nanoparticles was directly ascertained using this method. The nanoparticles of rosin maleic anhydride that were visible in the micrograph had an affinity for one another because they were noticeably stuck together.

The size of the particles ranged from spherical to irregular morphology and rough surface, and it was discovered that their morphology was non-homogeneous. The particle structure produced in the rosin maleic anhydride nanoparticles was proven to be in nano dimensions under high magnification. The majority of the rosin maleic anhydride nanoparticles had a spherical form, and their mean diameter ranged from 250 to 350 nm.

### 2.3. Inhibitory Activity of Rosin Maleic Anhydride Nanoparticles

**Figure 3:**
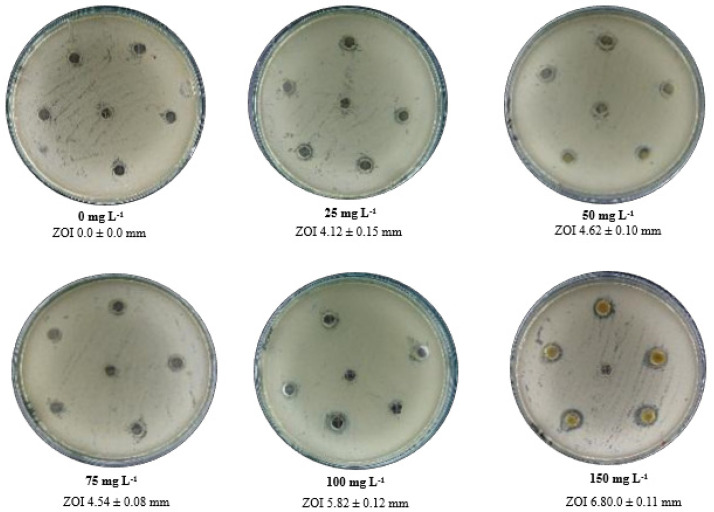
Different concentrations of rosin maleic anhydride nanoparticles tested for the antibacterial susceptibility of MRSE at 5 × 10^5^ cfu mL^−1^ and 35 °C for 24 h.

Rosin and rosin maleic anhydride nanoparticles’ inhibitory effects on *S. epidermidis* were investigated (Figure 3). In comparison to rosin, rosin maleic anhydride nanoparticles showed greater inhibitory effects when added in several quantities to MRSE, most notably at a concentration of 150 mg L^−1^ for the nanoparticles. These data analyses have demonstrated that rosin maleic anhydride nanoparticles only became more effective when MRSE is dosed with a higher concentration. For MRSE treated with rosin maleic anhydride nanoparticles, average ZOI data were also recorded. The examination of ZOI further clarified the relationship between rising rosin maleic anhydride nanoparticle concentration and rising ZOI, which is a result of rosin maleic anhydride nanoparticle action against MRSE. The ZOI ranged from 4.12 mm to 6.80 mm for the rosin maleic anhydride nanoparticles, which were generated at concentrations of 25 to 150 mg L^−1^.

**Figure 4:**
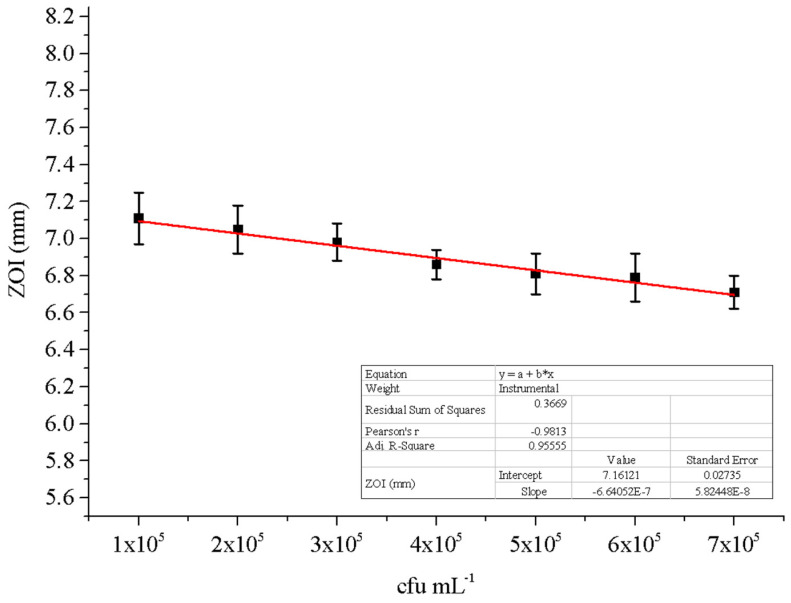
The effect of cfu mL^−1^ on the ZOI of MRSE at 150 mg L^−1^ of rosin maleic anhydride nanoparticles, after incubation at 35 °C for 24 h.

The response of 150 mg L^−1^ doses to MRSE concentrations ranging from 1 × 10^5^ to 7 × 10^5^ cfu mL^−1^ was studied (Figure 4). According to data that fit well linearly with an R^2^ coefficient of determination of 0.95, the ZOI gradually declines as the MRSE concentration increases. As a result, the effects of changing the cfu mL^−1^ of MRSE were predictable because it was discovered that a higher dosage of rosin maleic anhydride nanoparticles >150 mgL^−1^ should be needed for the efficient treatment of MRSE at higher numbers of bacterial cells.

**Figure 5:**
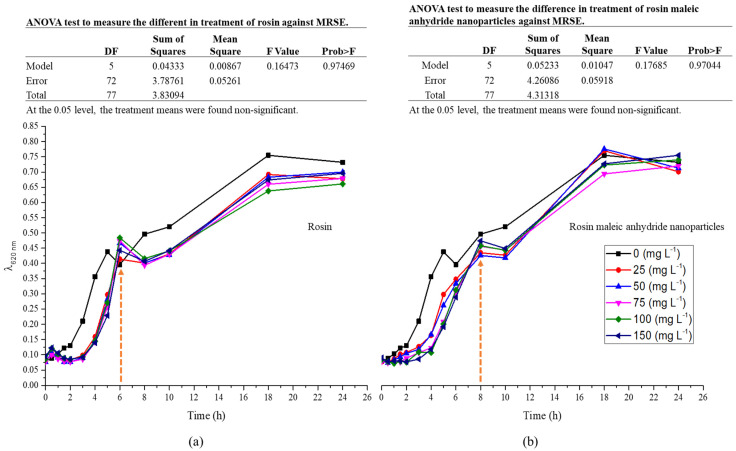
MRSE growth response to treatments: (**a**) rosin, (**b**) rosin maleic anhydride nanoparticles.

The pattern of growth between rosin and rosin maleic anhydride nanoparticles was explained by the growth curve of MRSE in a liquid medium (Figure 5a,b). However, increasing various rosin concentrations was seen to have a suppressive effect on MRSE growth (Figure 5a). The response of MRSE to changing the rosin content was not found to differ significantly (*p* < 0.05). Fast growth was experienced within the first six hours, but it then slowed down for six to eight hours before increasing again. Rosin inhibited growth throughout the growing period compared to the control medium, which contained no rosin.

Following the application of rosin maleic anhydride nanoparticles, the growth curve of MRSE was observed (Figure 5b). After being treated with various doses of rosin maleic anhydride nanoparticles, MRSE grew less slowly than the control. The MRSE, however, continued to rise significantly more quickly until 8 h. After this time, there was an 8–10 h growth halt. After 10 h, the growth’s gradual acceleration was seen. Rosin maleic anhydride nanoparticle-treated MRSE samples showed slower development over time compared to the control.

**Figure 6:**
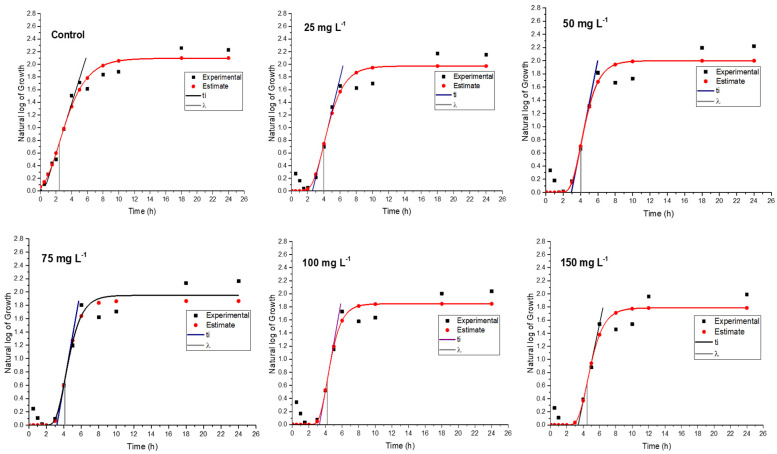
Experimental and predicted MRSE growth curve in response to rosin treatment.

**Figure 7:**
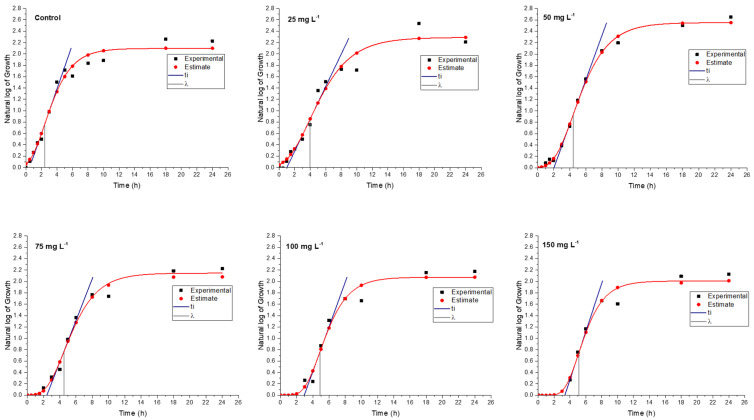
MRSE experimental and predicted growth curve in response to rosin maleic anhydride nanoparticle treatment.

**Figure 8:**
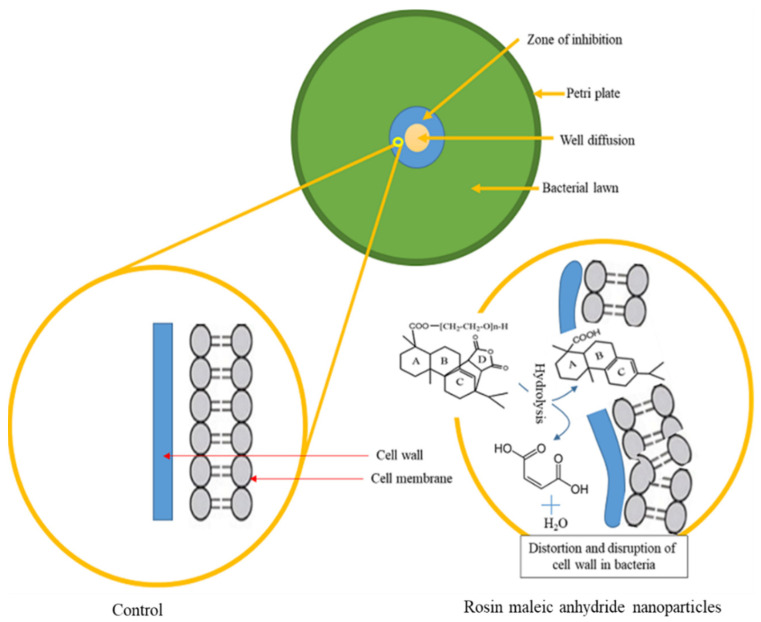
Rosin maleic anhydride nanoparticles’ proposed mode of action against MRSE.

Rosin maleic anhydride nanoparticles’ ability to stop the spread of contagious MRSE was enhanced. Rosin maleic anhydride nanoparticles were successful in stopping the growth from 6 to 8 h (Figure 5a) to 8 to 10 h (Figure 5b). It is crucial that rosin maleic anhydride nanoparticles work better with bacterial cells and disrupt intracellular metabolism. Consequently, over the course of treatment, this interaction has an effect on the delayed development and enhanced toxicity of rosin maleic anhydride nanoparticles to MRSE. Further analysis of growth data utilizing Gompertz kinetics was performed in order to more accurately estimate the MRSE’s growth response.

### 2.4. Gompertz Kinetics

In a medium containing various concentrations of rosin and rosin maleic anhydride nanoparticles, the bacterial culture of MRSE was developed for up to 24 h. To further forecast growth parameters from trend lines, the growth data were gathered and fitted into Gompertz kinetic Equation (1). Rosin maleic anhydride nanoparticles ranging in concentration from 25 to 150 mg L^−1^ were gradually introduced to the MRSE culture medium. The observed growth and projected growth from Gompertz kinetics in response to rosin (Figure 6) and rosin maleic anhydride nanoparticles are both explained graphically (Figure 7).

The range of predictions given by Gompertz kinetics for different growth estimations is reasonable. The majority of the data lies roughly on the Gompertz kinetics Equation (1) line. Due to interaction with the rosin surface, there is an abrupt increase in growth up to 6 h, which then stabilizes for the remaining 24 h. Therefore, 6 to 24 h are needed for the better activity of the rosin. The natural log values of the growth of MRSE are pushed from 2.1 to 1.8 over the period of growth by the rising rosin concentration. When MRSE was treated with rosin, the lag phase (*λ*) was observed. Therefore, extended values of *λ* clearly demonstrated the impact of rosin on impeding the growth of MRSE.

Through the synthesis of rosin maleic anhydride nanoparticles, this research compares and enhances the rosin’s activity. Figure 7 depicts the MRSE’s response to treatment with the rosin maleic anhydride nanoparticles in terms of growth. Although the curve followed a more gradual shift in growth, it was found that the growth patterns of MRSE treated with rosin maleic anhydride nanoparticles were comparable to those of MRSE treated with rosin. This can be seen by contrasting the values. The maximum value of the values was attained when the rosin maleic anhydride nanoparticles were present in the highest concentration. However, there was an apparent agreement between the experimental and Gompertz kinetics-estimated growth curves. As a result, it supported the fit of the Gompertz kinetic model to the development of MRSE in the presence of nanoparticles of rosin maleic anhydride.

According to the findings in Table 3, there was a difference between the trends for rosin and rosin maleic anhydride nanoparticles at the *µ_max_* (specific growth rate) of MRSE. The *µ_max_* was seen to increase 29.71% after MRSE received doses of rosin between 25 and 75 mg L^−1^, but this was followed by a drop of 17.91% when rosin dosages between 100 and 150 mg L^−1^ were used. When MRSE was treated with rosin maleic anhydride nanoparticles, the same trend was seen, with some variations. When MRSE was treated with rosin maleic anhydride nanoparticles, the *µ_max_* response was 32.54% greater.

In samples treated with rosin maleic anhydride nanoparticles, the *µ_max_* values were higher than the control values. *µ_max_* increased after being exposed to rosin maleic anhydride nanoparticles. The rosin maleic anhydride nanoparticles employed to combat MRSE made it very evident that the *µ_max_* values were lower than in samples treated with only rosin. Both rosin and rosin maleic anhydride nanoparticles exhibit a divergent trend in the *µ_max_* of the MRSE. *µ_max_* increased by 29.71% when the rosin dose was varied from 25 to 75 mg L^−1^, as was seen. However, it was shown that against 100 to 150 mg L^−1^ of rosin, there was a maximum drop of 17.91%. When MRSE was treated with rosin maleic anhydride nanoparticles, this trend changed. When MRSE was treated with rosin maleic anhydride nanoparticles under the same circumstances, *µ_max_* response was 32.54% higher. This shows that the rosin maleic anhydride nanoparticles’ harmful effects on MRSE were induced by enhanced cellular absorption and intracellular metabolism.

Data on the Gompertz kinetics of MRSE are shown in Table 3. The difference in growth parameters was caused by the treatment of rosin and rosin maleic anhydride nanoparticles. MRSE’s asymptotic growth (*A*) exhibited a declining tendency as the rosin concentration was increased. Values of *A* continued to be lower than control values. The changes in values of *A* likewise revealed a declining tendency in the case of rosin maleic anhydride nanoparticles. However, as compared to the control, the values of *A* were observed to be greater in the samples of MRSE treated with 25 and 50 mg L^−1^ rosin maleic anhydride nanoparticles.

The λ for MRSE was extended by rosin treatment. At rosin dosages of 25 to 50 mg L^−1^, the change in *λ* was 1.22%, and at dosages of 75 to 150 mg L^−1^, it was 4.22%. In this instance, rosin-treated MRSE had shorter λ values than those treated with rosin maleic anhydride nanoparticles. However, it was discovered that using rosin maleic anhydride nanoparticles had a greater influence on extending the λ. The response to doses of rosin maleic anhydride nanoparticles ranging from 25 to 50 mg L^−1^ and 75 to 150 mg L^−1^, respectively, showed λ values of 10.22% and 3.26%. Values of the λ decrease with increasing concentrations of rosin maleic anhydride nanoparticles. As a result, rosin maleic anhydride nanoparticles helped to slow down MRSE growth. Both the rosin and rosin maleic anhydride nanoparticles treatment lengthened the *λ* for MRSE development.

The growth curve’s curvature changes at the inflection point (*t_i_*), indicating that the maximum growth rate has been attained before it starts to decline. Therefore, in the case of rosin and rosin maleic anhydride nanoparticles, high values relative to the control explained the growth rates rising faster before the growth rate recessed. The interaction and support offered by the nanoparticles caused the inflection to be more curved in response to rapid growth. This demonstrated that rosin maleic anhydride nanoparticles successfully interact with MRSE and prolong the growth recession phase, i.e., *λ*, while reducing the time it takes to reach the *t_i_*.

## 3. Discussion

Infectious samples from hospitals frequently contain MRSE. Its increasing prevalence creates a significant challenge to investigate the novel design of more environmentally friendly and nanotechnology-based treatment alternatives. Different infectious clinical samples delivered to CMH Muzaffarabad hospital were found to have a prevalence of both MRSE (59.3%) and MRSS (40.62%) in the currently reported work (Table 1).

All seven antibiotics that were evaluated showed patterns of resistance to MRSE (Table 2). Despite the fact that six out of 38 MRSA isolates (15.78%) were vancomycin-resistant, the development and testing of the rosin maleic anhydride nanoparticles in the current research are justified. A unique technique for the treatment of MRSE that has been investigated is the use of rosin maleic anhydride nanoparticles. The multiple antibiotics resistance rate in our study was 56.3%, which is consistent with earlier published research [27]. According to that study, 65.43% of *S. epidermidis* with multidrug resistance had generalized antibiotic resistance. Furthermore, 82.07% of the 65.43% of those tested had a high level of vancomycin resistance. The most significant antibiotic class that is frequently used to treat MRSE is the glycol-peptide vancomycin. It is possible that the MRSE isolates examined in this study developed this resistance both while being treated in hospitals and as a result of excessive antibiotic dosages. A resistance gene is included in the MRSE on the plasmid. It is a known resistance gene that was potentially transferred to a non-resistant strain through the bacterial conjugation phenomenon, which is a typical method of horizontal gene transfer [28].

Nanoparticles derived from biopolymers or environmentally friendly sources are welcomed because the majority of these nanomaterials play a part in the defense against infection by the microorganisms to which they belong. A few different acids comprise rosin. Rosin is a natural insect repellent produced by plants. Recently, the development of antiviral drugs has exploited rosin and its derivatives [29]. Additionally, rosin soap has been created with virucidal properties [30]. Therefore, the literature provides strong support for the manufacture and application of rosin maleic anhydride nanoparticles. However, the novel idea illustrated here is the utilization of rosin maleic anhydride nanoparticles against MRSE. The rosin maleic anhydride nanoparticle size achieved in the current study ranged from 250 to 350 nm (Figure 2), which is consistent with a past study where it was revealed that rosin nanoparticles ranged in size from 100 to 200 nm [31]. It is known that rosin’s water solubility is increased by the maleic anhydride adduct [32]. As a result, using maleic anhydride to process nanoparticles in water as a universal solvent is simple and effective. Rosins, which include carboxylate groups that are negatively charged, aid in the creation of dispersible micelles and monomeric ions in water [33].

Increasing the concentration of the rosin maleic anhydride nanoparticles has proved to have an effect on MRSE through higher zones of inhibition (Figure 3 and Figure 4). Moreover, the prolonged λ and higher *µ_max_* delayed from 6 to 8 days for MRSE growth to reach the maximum after treatment with the rosin maleic anhydride nanoparticles (Figure 5a,b). This interesting fact is supported by earlier reports. A study of the effect of cerium oxide nanoparticles against *S. epidermidis* has shown that the ratio of live to dead cells did not show any difference that linked to a bacteriostatic effect rather than to an antibacterial effect.

Through greater zones of inhibition, the usage of rosin maleic anhydride nanoparticles at higher concentrations has demonstrated its effectiveness against MRSE (Figure 3 and Figure 4). In addition, after treatment with the rosin maleic anhydride nanoparticles, the maximum growth of the MRSE took 6 to 8 days to reach (Figure 5a,b). The prior reports provide support for this intriguing fact. According to a study, the effectiveness of cerium oxide nanoparticles against *S. epidermidis* was demonstrated by the lack of a variation in the ratio of live to dead cells, which was associated with a bacteriostatic impact rather than an antibacterial effect [34]. The effects of rosin maleic anhydride nanoparticles on the development parameters of MRSE, namely *A*, *t_i_*, and *µ_max_*, are displayed in Table 3 and Figure 7. Growth metrics give a precise insight into how rosin inhibits MRSE. One potential explanation for the bacteriostatic activity could be the resistance phenomenon brought on by the thickness of the cell wall, which affects the bactericidal and bacteriostatic effects of these rosin maleic anhydride nanoparticles. Moreover, ionized phosphoryl and carboxylate substituents of the macromolecules present on the outer cell envelope that is exposed to the extracellular environment provide bacterial cell surfaces with a net negative electrostatic charge [35]. Therefore, it is possible that the charge difference circumstances present at the time of treatment have a significant impact on the interaction between negatively charged rosin maleic anhydride nanoparticles and the negatively charged bacterial cell surface. In order to fully understand the impact of the potent antibacterial capabilities of the rosin maleic anhydride nanoparticles, more research will be needed.

The treatment of MRSE with rosin maleic anhydride nanoparticles was noted to have a strong bacteriostatic impact. As seen in Figure 8, the mechanism is based on a potential contact between the cell wall of MRSE and rosin maleic anhydride nanoparticles during experimentation. The MRSE’s cell wall is initially coated with rosin maleic anhydride nanoparticles. Due to the nanoparticles of rosin maleic anhydride, the cell wall was possibly distorted and disrupted. The hydrolytic activity of membrane-embedded proteins may also have degraded the rosin maleic anhydride nanoparticles following treatment with MRSE. The hydrolytic activity of cell membrane-embedded proteins, which remove maleic anhydride from the rosin of nanoparticles and convert it to maleic acid and water, may further intensify this impact. Cell wall integrity may have been weakened in these acidic conditions; as a result, when the cell wall comes into contact with rosin maleic anhydride nanoparticles, it is first deformed and then gradually disrupted. This event might lessen the cell wall’s ability to fend against nanoparticles made of rosin maleic anhydride. The rosin maleic anhydride nanoparticles produced as a result of this procedure have bacteriostatic and inhibitory effects against MRSE.

Maleic anhydride’s solubility, emulsification, and surface characteristics are also well-known in the literature [36]. Therefore, the rosin activity may be more effective against the rupture and distortion of cell walls as a result of these characteristics of maleic anhydride. This theory that MRSE is stressed due to the bacteriostatic effect of the rosin maleic anhydride nanoparticles over an extended length of time might be supported by the difference in the growth of MRSE, as shown in Figure 7. In order to address resistance in MRSE, this work establishes the rationale for using rosin in the form of rosin maleic anhydride nanoparticles.

## 4. Materials and Methods

### 4.1. Sample Collection

The numerous clinical samples were obtained on Petri dishes from the Microbiology Laboratory of the Combined Military Hospital (CMH), Muzaffarabad, Azad Jammu and Kashmir, Pakistan. The 4 different infectious specimen types—urine, blood, ear swabs, and pus—were collected. For further testing of *S. epidermidis*, these samples were brought to the Biotechnology Laboratory at the University of Azad Jammu and Kashmir. A *Pinus roxburghii* plant from the Muzaffarabad forest was used to harvest rosin. Before being used, the rosin was heated and forced through a sieve to remove any impregnated woody debris.

### 4.2. Culturing Conditions

The samples were taken on nutritional broth and incubated for 24 h at 37 °C. Following the optical density (O.D.) data at a wavelength of 620 nm, the increase was monitored for 18 h. A full loop of the inoculum was taken in sterile conditions in a laminar flow and smeared over the agar Petri dishes at an OD of 0.8. For the next 24 h, the Petri dishes were incubated at 37 °C. Carefully, one microbial colony was selected from the culture, streaked on mannitol salt agar, and then re-incubated at 37 °C for 24 h in order to identify and isolate *S. epidermidis*.

### 4.3. Isolation and Identification

**Figure 9:**
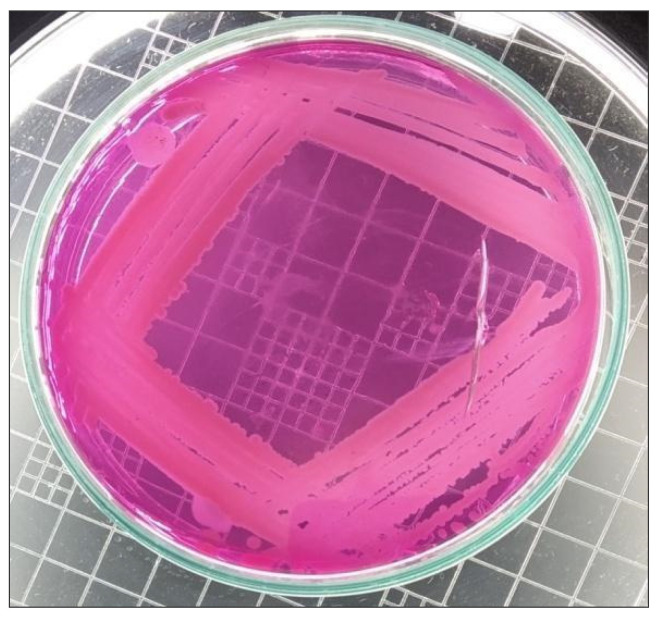
Growth of *S. epidermidis* on MSA.

The biochemical tests, such as catalase and coagulase [37], and colony appearance on Mannitol Salt Agar (MSA), were used to determine the identity of the cultured isolates as *S. epidermidis*. Further cultivation of the isolated *S. epidermidis* on certain MSA media allowed for the isolation and identification of *S. epidermidis*. *S. epidermidis* was streaked on MSA, incubated at 37 °C for 24 h to determine colony appearance, and identified when a pink colony appeared on MSA [38], as shown in Figure 9.

The sequence analysis of the 16S ribosomal RNA gene was used for the molecular identification of the *S. epidermidis* to confirm the bacterial species. Using the phenol-chloroform technique, DNA was isolated from a single colony of the bacterium that was taken from the MSA plate [39]. The 16S rRNA gene was amplified using the universal primers 27F (5′-AGAGTTTGATCCTGGCTCAG-3′) and 1492R (5′-TACGGYTACCTTGTTACGACTT-3′) [40]. The National Library of Medicine, 8600 Rockville Pike, Bethesda, MD, USA (https://blast.ncbi.nlm.nih.gov/Blast.cgi, accessed on 25 March 2022) offers the basic local alignment search tool BLASTN, which was used to evaluate the 16S rRNA gene sequence.

### 4.4. Synthesis of Rosin Maleic Anhydride Nanoparticles

In order to synthesize rosin maleic anhydride, the established literature was followed [41]. Maleic anhydride, zinc dust, and polyethylene glycol (PEG) were utilized. Rosin was heated and kept at 270 °C in a silicon oil bath using a double-necked round-bottom flask with a condenser. The hot rosin was mixed with 9.90 g of PEG and 0.125 g of zinc dust as catalysts. To create rosin maleic anhydride, 1.61 g of maleic anhydride was introduced at a temperature of 240 °C, cooled to 160 °C, and then the reaction continued further for 6 h [32]. Additionally, the rosin maleic anhydride was employed to create nanoparticles using the previously mentioned cold water dispersion method [31]. An amount of 1 mL of the rosin maleic anhydride solution was added to 19 mL of the 4 °C cold water. For 12 h, this mixture was vigorously mixed. When the rosin maleic anhydride was turned into a homogenous, slightly turbid solution of nanoparticles, the reaction was terminated.

### 4.5. Field Emission Scanning Electron Microscopy

By using Field Emission Scanning Electron Microscopy, the surface shape and size of the rosin maleic anhydride nanoparticles were determined (FESEM, Model JEOL JSM-6390 TESCAN Vega 3 LMU-variable pressure Scanning Electron Microscope). The preserved samples were deposited on the inserts, and then gold or palladium was sputtered onto them. The samples were then examined after the procedure had been stopped for 4 min at 20 mA. Using a free software called ImageJ, which can be downloaded from the internet at https://imagej.nih.gov/ij, accessed on 1 March 2022, the size of the nanoparticles was calculated from the images.

### 4.6. Antibiogram Profiling

The Biotechnology Laboratory at the University of Azad Jammu and Kashmir in Muzaffarabad conducted drug susceptibility testing. *S. epidermidis* isolates were cultured on enrichment media MSA for 24 h at 37 °C. The disk-diffusion method was used to assess the susceptibility of antimicrobials on Mueller Hinton Agar (MHA). The Clinical and Laboratory Standard Institute, Pennsylvania, USA, guidelines were followed while evaluating drug susceptibility [42].

All *S. epidermidis* isolates were subjected to disc diffusion antimicrobial drug susceptibility testing on agar plates. *S. epidermidis* colonies were removed from the pure culture and transferred to a tube containing 5 mL of distilled water. The mixture was then gently blended until a homogeneous suspension was achieved. The bacteria covered the whole surface of the MHA after being dispersed by the suspension mixture. The following antibiotic concentrations were tested against *S. epidermidis*: oxacillin (1 µg), amoxicillin (25 µg), cefoxitin (30 µg), vancomycin (30 µg), piperacillin/tazobactam (110 µg), cephradine (30 µg), and fosfomycin (50 µg) (Table 4). Following this incubation period, oxacillin-resistant strains of *S. epidermidis* were reported in accordance with the zone of inhibition (ZOI) specifications outlined in the CLSI map 2013. Utilizing the free, online-available AntibiogramJ software (https://sourceforge.net/projects/antibiogramj/, accessed on 1 September 2022), the ZOI of *S. epidermidis* was calculated [43]. To analyze the antibiogram profile for *S. epidermidis*, the ZOI values to determine the resistance, intermediate, and sensitivity were used. Oxacillin-resistant *S. epidermidis* is known as methicillin-resistant *S. epidermidis* (MRSE), whereas oxacillin-sensitive S. epidermidis is known as methicillin-sensitive *S. epidermidis* (MSSE).

The MRSE isolate CMH71 was used in this study’s additional examination to examine the efficiency of rosin and rosin maleic anhydride nanoparticles. The response of the MRSE to different concentrations of rosin and rosin maleic anhydride nanoparticles was evaluated using a well-diffusion technique. Each experiment’s ZOI was recorded. The concentrations of rosin and rosin maleic anhydride nanoparticles utilized were 25, 50, 75, 100, and 150 mg L^−1^. The serial dilution procedure was also used to create MRSE cell concentrations ranging from 1 × 10^5^ to 7 × 10^5^ cfu mL^−1^. Each cfu mL^−1^ value was assessed in comparison to the rosin maleic anhydride concentration that had the maximum activity.

### 4.7. Gompertz Growth Kinetics

After being exposed to ultraviolet rays in a laminar flow for 30 to 60 min, rosin and rosin nanoparticles were sterilized. After being treated with rosin and rosin maleic anhydride nanoparticles, the growth of the MRSE was observed in a continuous batch culture being run in a 1 L flask. The growth was also programmed to be monitored for up to 24 h at regular intervals. At doses of 25, 50, 75, 100, 125, and 150 mg L^−1^, rosin and rosin maleic anhydride nanoparticles were evaluated, and the growth response of MRSE was determined by observing the OD at λ_620 nm_. The continuous batch culture was carried out under recommended pH (5.5) and temperature (37 °C) settings. An amount of 10 mL of the activated MRSE culture and 0.1 mL of the rosin and rosin maleic anhydride nanoparticles solution (10 times the diluted to × 10^3^ times the original level) were added separately to the culturing flask. Then, for 24 h at 37 °C, each flask was placed on the shaking incubator (IRMECO Gmbh, Lütjensee, Germany) at 120 rpm. The sample’s OD at λ_620 nm_ was then measured using a T60 UV-Visible spectrophotometer (PG Instruments, Lutterworth, UK). Bacterial growth or inhibition is directly correlated with an increase or reduction in the culture’s OD value, respectively. The antibacterial and bacteriostatic activity of rosin and rosin maleic anhydride nanoparticles was examined using the MRSE growth data. The Gompertz growth kinetics Equation (1) was fitted to the tabular growth data after it had been log converted [45].

(1) $$A\;exp\;exp\;\left\{-exp\left[\frac{\mu_{max}e}{A}\left(\lambda -t\right)+1\right]\right\}\;$$

The shift in growth parameters caused by the MRSE’s growth in response to rosin and rosin maleic anhydride was determined from Equation (1) as the specific microbial growth rate (*μ_max_*), lag time (*λ*), log maximum growth (*A*), and curvature of the curve t taken into account as *t_i_*.

### 4.8. Statistical Analysis

Statistics were used to compare the means of the treatments with rosin and rosin maleic anhydride nanoparticles for growth inhibition. The variance within the treatment groups was examined using a one-way analysis of variance (ANOVA) at the *F*-test significance level, which was set at *p* < 0.05. The Tukey post hoc test was utilized to determine the significant difference in the means of pairs of rosin and rosin maleic anhydride nanoparticle treatment groups if the variance in the data was determined to be significant. Through the OriginPro version 9.0.0 statistical program (OriginLab Corporation, Northampton, MA, USA), the ANOVA test was conducted on the sample data.

## 5. Conclusions

The study’s findings support the use of rosin maleic anhydride nanoparticles, which are suggested and proven to be effective treatments for MRSE. Clinical infectious sample MRSE prevalence was found to be 59.37% (38/64) in all samples. MRSE’s resistance to the main antibiotics used to treat this strain was anticipated by antibiogram profiling. The rosin maleic anhydride-derived nanoparticles, with an average size of 250 to 350 nm, were effectively produced as a solution to this issue. The growth time was increased from 6 to 8 h by the rosin maleic anhydride nanoparticles. Additionally, the rosin maleic anhydride nanoparticles demonstrated a successful method for enhancing the bacteriostatic and inhibitory efficacy against MRSE. Rosin maleic anhydride nanoparticles were more effectively metabolized in MRSE than rosin, which was corroborated by data on the lengthened lag phase (*λ*) and reduced specific growth rate (*μ_max_*). Low *μ_max_* is associated with greater nanoparticle digestion when compared to rosin, which increases the toxicity to MRSE, likely through mechanisms of cell wall breakage and deformation. This paper offers a thorough analysis of the utilization of plant-based bioresources in therapeutic settings to combat the growing problem of pathogen drug resistance.

## Figures and Tables

**Table 1:** Distribution of MRSE and MSSE based on clinical specimens.

Source of Specimen	Total (*n* = 64)	MSSE (*n* = 26)	MRSE (*n* = 38)
Pus	27	15	12
Ear swabs	03	-	03
Urine	27	11	16
Blood	07	-	07

**Table 2:** The numbers and percentages of MSSE and MRSE for different antibiotics.

Antibiotic	*S. epidermidis* (*n =* 64) *			
	MSSE (*n* = 26) **		MRSE (*n* = 38) **	
	No.	%	No.	%
Vancomycin	12	48.21	6	15.78
Piperacillin/Tazobactam	24	94.74	2	5.26
Amoxicillin	5	21.05	30	78.94
Oxacillin	10	42.11	21	55.26
Cephradine	20	78.95	8	21.05
Cefoxitin	6	26.32	28	73.68
Fosfomycine	3	10.53	34	89.47

* The total number of S. epidermidis isolates that demonstrated the evaluated antibiotic sensitivity (N). *^,^ ** Subsample size (n) is displayed as the sum of the MSSE and MRSE counts separately.

**Table 3:** Gompertz kinetics of MRSE culture treated with rosin and rosin maleic anhydride nanoparticles.

Composition	Conc. (mg L^−1^)	Asymptotic Growth *A*	Specific Growth Rate *µ_max_*	Lag Phase *λ*	Inflection Point *t_i_*	*R* ^2^
Rosin	0	2.101	0.393	0.773	2.448	0.928
	25	1.973	0.525	0.726	3.963	0.938
	50	1.998	0.658	0.735	4.050	0.938
	75	1.864	0.747	0.686	4.117	0.944
	100	1.846	0.720	0.679	4.210	0.954
	150	1.785	0.591	0.657	4.504	0.947
Rosin maleic anhydride nanoparticles	0	2.101	0.393	0.773	2.448	0.977
	25	2.292	0.286	0.843	3.958	0.972
	50	2.551	0.390	0.939	4.439	0.996
	75	2.080	0.366	0.765	4.496	0.982
	100	2.074	0.393	0.763	4.884	0.981
	150	2.012	0.424	0.740	5.102	0.985

**Table 4:** Criteria for the analysis of antibiotics sensitivity for *S. epidermidis* [42,44].

Antibiotic	Code	Disk Potency	Zone of Inhibition (mm)		
			Resistant	Intermediate	Sensitive
Cephradine	CE	30 µg	≤15mm	16–17 mm	≥18 mm
Cefoxitin	FOX	30 µg	≤21 mm	-	≥22 mm
Vancomycin	VA	30 µg	≤14mm	15–16mm	≥17 mm
Oxacillin	OX	1 µg	≤11 mm	12–13 mm	≥14 mm
Amoxicillin	AML	25 µg	≤19 mm	-	≥20 mm
Fosfomycin	FOS	50 µg	≤11 mm	12–15mm	≥16 mm
Pipracilin/Tazobactam	TZP	110 µg	≤16 mm	-	≥17 mm

## Data Availability

Not applicable.
